# Collective Trauma and Mental Health in Adolescents: A Retrospective Cohort Study of the Effects of Retraumatization

**DOI:** 10.3389/fpsyt.2021.682041

**Published:** 2021-06-25

**Authors:** Hannah Pazderka, Matthew R. G. Brown, Vincent I. O. Agyapong, Andrew James Greenshaw, Caroline Beth McDonald-Harker, Shannon Noble, Monica Mankowski, Bonnie Lee, Julie L. Drolet, Joy Omeje, Pamela Brett-MacLean, Deborah Terry Kitching, Peter H. Silverstone

**Affiliations:** ^1^Department of Psychiatry, University of Alberta, Edmonton, AB, Canada; ^2^Department of Computing Science, University of Alberta, Edmonton, AB, Canada; ^3^Department of Sociology and Anthropology, Mount Royal University, Calgary, AB, Canada; ^4^Fort McMurray Public School District, Fort McMurray, AB, Canada; ^5^Fort McMurray Catholic School District, Fort McMurray, AB, Canada; ^6^Faculty of Health Sciences, University of Lethbridge, Lethbridge, AB, Canada; ^7^Faculty of Social Work, University of Calgary, Edmonton, AB, Canada

**Keywords:** collective trauma, retraumatization, post-traumatic stress disorder, adolescent, trauma informed practice, stress contagion, sexual abuse trauma

## Abstract

In the wake of the massive Canadian wildfire of May 2016 in the area of Fort McMurray Alberta, we observed increased rates of mental health problems, particularly post-traumatic stress disorder (PTSD), in school-aged adolescents (ages 11–19). Surprisingly, we did not see these rates decline over the 3.5-year follow-up period. Additionally, our research suggested that the impact of this mass incident resulted in other unanticipated effects, including the finding that children who were not present for and relatively unaffected by the wildfire showed a similar PTSD symptom profile to children more directly involved, suggesting some degree of spillover or stress contagion. A potential explanation for these high rates in individuals who were not present could be undiagnosed retraumatization in some of the students. To investigate this possibility, we compared two groups of students: those who reported the wildfire as their most significant trauma (*n* = 740) and those who had their most significant trauma prior to the wildfire (*n* = 295). Those with significant pre-existing trauma had significantly higher rates of both depression and PTSD symptoms, although, unexpectedly the groups exhibited no differences in anxiety level. Taken together, this evidence suggests retraumatization is both longer-lasting and more widespread than might be predicted on a case-by-case basis, suggesting the need to reconceptualize the role of past trauma history in present symptomatology. These findings point to the need to recognize that crises instigated by natural disasters are mass phenomena which expose those involved to numerous unanticipated risks. New trauma-informed treatment approaches are required that incorporate sensitivity to the collective impact of mass crises, and recognize the risk of poorer long-term mental health outcomes for those who experienced trauma in the past.

## Introduction

Mass trauma events such as the Canadian wildfire of May 2016 in the area of Fort McMurray Alberta provide a unique opportunity to examine how an entire population reacts to a traumatic event, and different factors that might affect the risk profile of individuals in these circumstances. In May 2016, a severe wildfire burned 590,000 hectares and caused the mass evacuation of all 88,000 residents of this remote northern Canadian, energy industry-focused town. In this study, we sought to examine whether adolescents who had undergone previous significant trauma prior to the wildfire would be impacted differently than those undergoing trauma for the first time.

It is reasonable to assume that previous trauma could be associated with heightened reactivity to a new trauma event, due to what has been termed retraumatization. In behavioral terms, chronic maltreatment has been shown to hinder maturation, increasing risk for PTSD symptoms (1), as well as internalizing and externalizing behaviors (2). This is possibly due to structural and/or functional alterations in the brain (3) occurring as a product of prolonged hypothalamic pituitary adrenal (HPA) axis activation (4); accordingly, it has been posited that permanent sensitization of this pathway is a consequence of childhood abuse (5). Structural changes in the amygdala have recently been confirmed in human subjects (6). Given these neural changes, retraumatization—a condition in which an individual with pre-existing trauma is triggered by a new stressor, presumably responding more quickly or intensely—is a potential outcome. Indeed, a number of studies appear to confirm that an earlier trauma carries repercussions for how one reacts to later trauma (7, 8), potentially suggesting changes in stress reactivity (5). What has been less recognized is the propensity for these kinds of underlying issues to inform how one responds in a mass trauma situation, in which it is unclear exactly which individuals are at increased risk for retraumatization.

In the present study, we used data from adolescents who experienced the Fort McMurray wildfire to compare the rates of mental health conditions in individuals with, or without, significant trauma prior to the wildfire. Although, a thorough examination of Adverse Childhood Events (ACEs) would have been ideal, our current analysis was limited to retrospective self-reports of whether or not the individuals had experienced some worse self-reported trauma prior to the wildfire. Aside from the mental health diagnostic information, we also examined increased propensity for recent suicidal ideation and rates of suicide attempts overall, as previous research has posited particularly high rates of suicidality amongst disaster survivors (9). Thus, the goal of the present analysis was to compare negative mental health symptomatology between the two groups. We hypothesized that adolescents who had experienced prior trauma would show poorer mental health, specifically higher rates of anxiety, depression, and post-traumatic stress disorder (PTSD), and higher rates of recent suicidal ideation and lifetime suicide attempts.

## Methods

### Survey Administration Procedure

The full mental health survey was developed by the Fort McMurray school systems in conjunction with the research team to evaluate their post-wildfire programming. They administered all aspects of data collection, as per their standard procedures and policies. Accordingly, all students enrolled in either junior or senior high schools in both Public and Catholic School Districts in Fort McMurray were invited to participate in data collection, although, parents could opt their child(ren) out if desired (fewer than 10 exemptions were requested). The total survey battery consisted of 96 questions [for full description of measures see Table 1; a detailed question-by-question breakdown has been published elsewhere (10)]. Use of all survey methods and materials was approved by the Health Research Ethics Boards (ethics protocol number Pro00072669) at the University of Alberta, Canada. Students completed multiple mental health questionnaires each year for a 3-year period starting in November 2017. Because the Fort McMurray school boards mandated that students complete the questionnaires anonymously (procedure described in detail below), there was no way to track individuals from year-to-year, so data analysis for this study was confined to a cross-sectional analysis. Accordingly, the data in this study represent the third round of data collection (gathered approximately 3.5 years post-wildfire), selected on the conservative premise that this timeframe would give individuals the maximum amount of time for recovery.

**Table 1:** List of measures administered to junior and senior high students after Fort McMurray wildfire (2017–2019).

**Instrument name**	**Author(s)**	**Domain measured**	**Format**
Ft. McMurray demographics questionnaire	Brown et al. (10)	Gathers basic demographic information, including name, age, sex, grade, school, and homelessness.	Seven items with pre-specified choices.
Impact of fire questionnaire	Brown et al. (10)	Custom questionnaire designed to assess impact of the 2016 wildfire.	Six items: 4 y/n items measure proximity and impact of fire, plus two assess school affiliation.
Patient Health Questionnaire, Adolescent version (PHQ-A)	Johnson et al. (11)	Assesses depression symptom severity (past 2 weeks) as well as suicidal ideation (past month) and history of previous suicide attempts (lifetime). Probable depression is defined as having a PHQ-A score of 11 or more (12).	Nine depression items measured on a 4 point Likert scale assess frequency of symptoms, plus 2 y/n suicidality questions; total score from 0 to 27 (depression); suicide score is an additional 2 points (scored separately).
Hospital Anxiety and Depression Scale (HADS), anxiety-related questions only	Zigmond and Snaith (13)	Assesses symptoms of anxiety in the past week. Probable anxiety is defined as having a HADS score of 11 or more (14).	Seven items on a 4 point Likert scale, with items for both frequency and severity; score from 0 to 21
Child PTSD Symptom Scale (CPSS)	Foa et al. (15)	Assesses PTSD symptoms. Symptoms grouped into three subcategories: re-experiencing, avoidance, and hyperarousal. Two additional items query about the most distressing event the respondent has experienced and when it occurred. Probable PTSD is defined by a CPSS score of 16 or more (16).	Nineteen questions: 17 items measured on a 4 point Likert scale assess frequency of symptoms from 0 to 51; additional two items offer pre-specified choices.
CRAFFT questionnaire (CRAFFT)	Knight et al. (17)	Assesses symptoms of alcohol and substance misuse over the past 12 months. Probable alcohol/substance use disorder was defined as having a CRAFFT score of 2 or more (17, 18).	Nine items, scored y/n from 0 to 9.
Tobacco use items	Brown et al. (10)	Items added to CRAFFT regarding tobacco and smokeless tobacco use.	Two items, scored y/n from 0 to 2.
Rosenberg self-esteem scale	Rosenberg (19)	Assesses self-esteem, with items reflecting agreement with how well the concepts describe the respondent.	Ten items on a 4 point Likert scale measuring agreement; provides a score from 0 to 30.
Kidscreen questionnaire (Kidscreen-10)	Ravens-Sieberer et al. (20)	Assesses quality of life in terms of how the respondent has been feeling, opportunities for recreation, and socialization	Eleven items on a 5 point Likert scale measuring frequency; provides a score from 0 to 44.
Child and Youth Resilience Measure (CYRM-12)	Liebenberg et al. (21)	Assesses the resources available to individuals that may bolster their resilience.	Twelve items on a 5 point Likert scale measuring agreement; provides a score from 12 to 60^†^.

† *Scores for these scales recoded to maintain consistency amongst study measures*.

Data collection occurred over a 9-day period in November 2019, during which all 6 school sites were visited by the data collection team. Students were excused from normal classroom activities in order to complete the questionnaires, with no penalty for non-participation. Students were seated in a classroom together, fitted with separate computer monitors (either laptops or desktops, depending upon the school); these were either the school's computer room, a library, or in some instances a convenient classroom, and they seated on average 25–50 students. A script explaining why data were being gathered, outlining expectations regarding their behavior during data collection, and that thanked them for their participation, was read to the students by a member of the data collection team. Students were asked to try to answer every question, but informed that they could skip any questions that they did not understand, or that made them feel uncomfortable. Students were ensured their answers would be kept anonymous, and were not asked for their names, nor were they assigned unique identifiers (ID numbers). They were asked to fill out the questionnaires individually, without consulting or discussing with those around them, however staff were available to answer questions in the event of any questions, or in the event of any technical difficulties. Sessions averaged 20–25 min, although, students were given as much time as they required to complete the questionnaires.

### Data Analysis

To assess effects of collective traumatization on long-lasting PTSD and related mental health characteristics, this retrospective cohort study specifically compared two groups: those exposed only to the collective trauma (wildfire), and those with a history of previous childhood trauma. A two-tailed, independent-samples *t*-test was conducted to compare the mental health variables described above in the prior trauma group with those who listed the wildfire as their most traumatic event (no previous trauma). All means were tested at the 95% probability level, with pairwise deletion used for missing data. Analyses were completed using IBM SPSS Statistics-v26 software.

**Figure 1:**
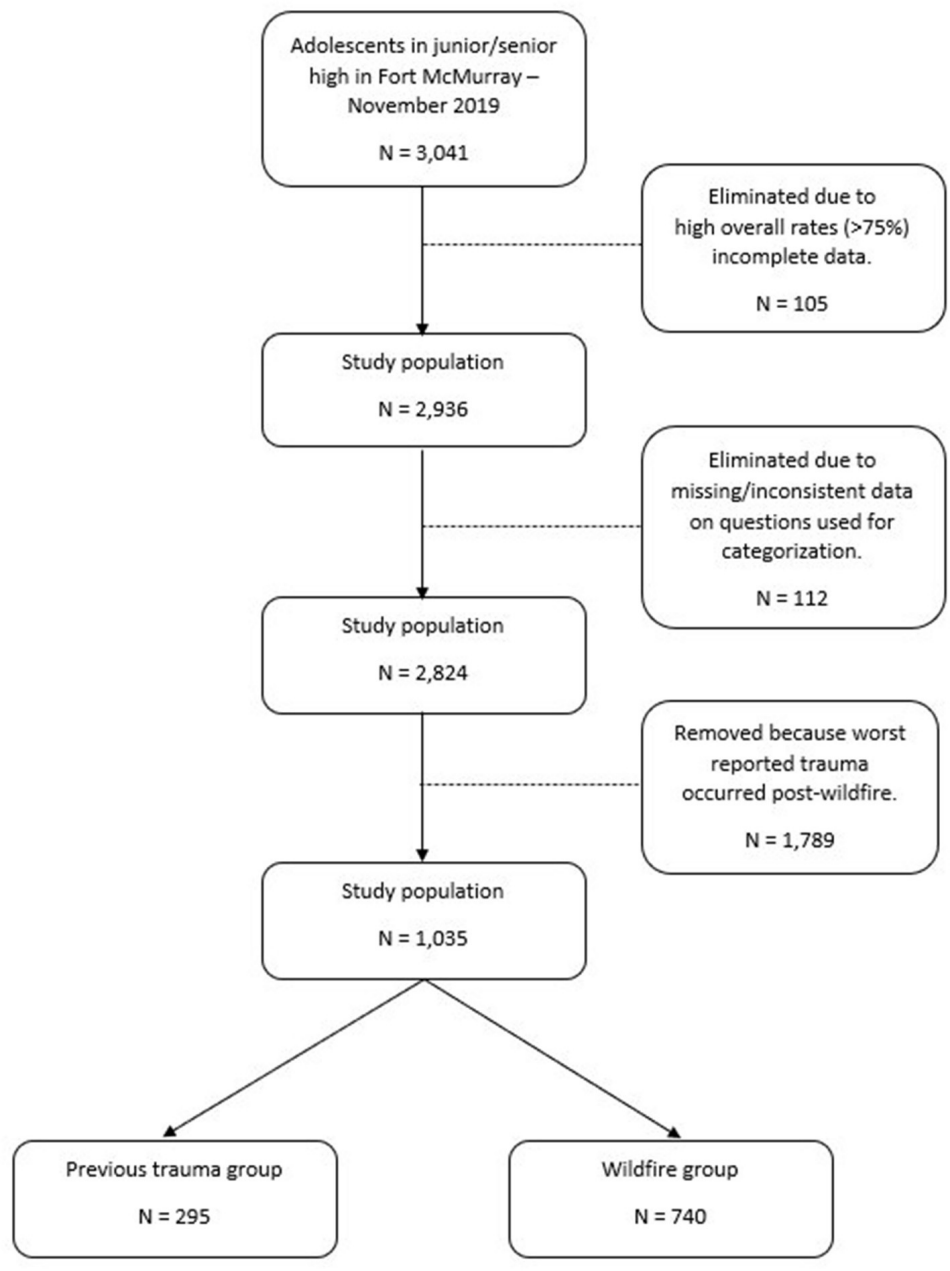
Subject flow diagram illustrating number of individuals in each group.

Fort McMurray students who were available and willing to participate in the survey comprised 3,041 youth aged 11–19 years, who completed the questionnaires. Data from 105 students (3.5%) were eliminated due to exceedingly low rates of questionnaire item responses, leaving an overall sample size of 2,936. A detailed subject flow diagram is presented in Figure 1.

Because all students present between grades 7 and 12 participated (i.e., the entire junior/high school population of Fort McMurray present at the time of testing), no sample size calculation was performed. Although, there were no specified exclusion criteria for the study, individuals not attending class at the time were not included. Additionally, adolescents of that age going to school in rural areas outside the Fort McMurray townsite were also not included.

### Mental Health Questionnaires of Interest

For the purposes of this investigation, the following measures were examined:

- *Depression*—Patient Health Questionnaire, Adolescent version (PHQ-A, 11 questions) (11): Assesses symptoms of depression. Scores for severity range from 0 to 27.
- *Suicidality*—Patient Health Questionnaire, Adolescent version (PHQ-A, 2 questions). In addition to the depression items, the PHQ-A specifically queries regarding suicidal thoughts, as reflected in 2 y/n items: “*Over the past 2 weeks, how often have you been bothered by any of the following problems? Thoughts that you would be better off dead, or of hurting yourself in some way?”* and “*Has there been a time in the past month when you have had serious thoughts about ending your life?”* These items were presented only if the respondent acknowledged thoughts regarding hurting him/herself or feeling they would be better off dead on at least several occasions per week. Scores for these items were coded 0 = No, 1 = Yes.
- *Anxiety*—Hospital Anxiety and Depression Scale (HADS, 7 questions; Anxiety items only) (13): Assesses symptoms of anxiety; scores for anxiety symptom severity range from 0 to 21.
- *PTSD*—Child PTSD Symptom Scale (CPSS, 19 questions) (15): Assesses symptoms of post-traumatic stress disorder (PTSD); scores of PTSD symptom severity range from 0 to 51. Scores can also be grouped into three subcategories: re-experiencing, avoidance, and hyperarousal.

Individual items were also recoded where necessary such that a higher score reflected increased symptom severity. Where necessary, measures were recoded such that the base score for the Likert scale was 0 (reflecting absence of symptoms), for consistency between questionnaires.

## Results

To examine effects of previous traumatization on reaction to the wildfire, students were selected if they indicated that the wildfire was the worst trauma they had experienced (“wildfire group”), or if they indicated that they had experienced a worse trauma prior to the wildfire (“prior trauma” group). This information was assessed using two questionnaire items: “*Please select the most distressing event you have experienced”* and “*How long has it been since the event from the previous question.”*

Notably, nearly two-thirds (1789; 63.3%) of the total sample was excluded at this stage because they reported their most significant trauma had occurred in the time period *following* the wildfire. With this group excluded, the study sample consisted of 295 students in the “prior trauma” group and 740 students in the “wildfire” group (where this was their most significant trauma). Thus, of the 1,035 students who had reported a traumatic event up until and including the wildfire, almost a third (28.5%) had been previously traumatized.

### Characteristics of the Study Sample

Demographic characteristics of the 1,035 participants were as follows. Gender identity analysis corresponded to an approximately equal binary distribution, with 47.1% identifying as female, 47.7% as male, in addition 2.4% identified as other, and 2.6% preferred not to say. Age ranged from 11 to 19; mean (SD) age was 14.19 (1.78), with the median being 14. Junior high (grades 7–9) comprised 56.1% of the sample), with the remaining 43.9% in high school (grades 10–12), with the median being grade 9.

Exposure to wildfire was assessed using the following four y/n items, which were summed to generate a “total exposure” score. Students reported being in or near Fort McMurray at the time of the wildfire comprised 955 (92.4%). Among these students, 964 (93.3%) had to evacuate because of the fire but only 830 (80.2%) reported actually seeing the wildfire. Of these students, 133 (12.9%) reported that their home was destroyed. For these four items, students on average scored 2.79 (0.895), with the median being 3.

Of the prior trauma group, 107 (36.3%) reported that their worst trauma was the death of someone close to them; 36 (12.2%) reported it was a sexual assault; 22 (7.5%) reported that it was an injury they had suffered; and 6 (2%) said it was a physical assault. Another 124 (42%) did not specify the nature of the trauma. Over two-thirds of this group (68.5%) reported that that trauma had occurred between 6 and 10 years prior to completing the survey, while the remainder (31.5%) reported it was over 11 years prior.

### Group Comparisons

Comparing the two groups (see Table 2), individuals in the prior trauma group were significantly more likely to be male than those in the wildfire group (58.2 vs. 47.3%; χ^2^ (1) = 9.41, *p* = 0.00; all comparisons presented as prior trauma vs. wildfire, respectively). They were also 6 months older on average (M [SD]) (14.52 [1.76] vs. 14.04 [1.77]; *t*_1032_ = 3.97, *p* < 0.01). The prior trauma group experienced lower mean levels of exposure to the wildfire, and the overall variability in their scores was much higher (2.24 [1.26] vs. 3.01 [0.57]; *t*_341_ = −10.01, *p* < 0.01).

**Table 2:** Independent *t*-test comparison of prior trauma group (*n* = 295) vs. wildfire alone (*n* = 740) conditions.

	**Previous trauma Gp**			**Wildfire Gp**			***t*-value**
	***n***	**M**	**SD**	***n***	**M**	**SD**	
**Demographic variables**							
Sex (male)	273	58.2%	0.49	708	47.3%	0.50	3.09^**^
Age	295	14.52	1.76	739	14.04	1.77	3.97^**^
Exposure to wildfire	294	2.24	1.26	793	3.01	0.57	−10.01^**^
**Mental Health variables**							
Anxiety	292	7.29	5.00	738	7.62	4.62	ns
Depression	291	8.68	7.02	733	7.40	5.95	2.74^**^
PTSD	289	12.97	12.48	731	10.50	10.24	2.99^**^
–re-experiencing	294	3.07	3.72	735	2.23	2.96	3.48^**^
–avoidance	292	5.05	5.45	733	3.78	4.53	3.53^**^
–hyperarousal	290	4.80	4.31	732	4.51	3.96	ns
Serious thoughts of suicide – past month^1^	95	0.53	0.50	168	0.46	0.50	ns
Previous suicide attempt – lifetime^1^	94	0.37	0.49	167	0.27	0.45	1.69^†^

*M, mean; SD, standard deviation*.

† *0.05> p <0.10*.

** *p < 0.01*.

1 *Suicide questions coded as: 0 = no, 1 = yes*.

In terms of the mental health variables, there was a significant between-groups difference for both Depression (*t*_463_ = 2.74, *p* < 0.01) and PTSD (*t*_449_ = 2.99, *p* < 0.01), with students who reported previous trauma showing elevated rates on both measures. Results also indicated a an increased value of lifetime suicide risk for the prior trauma group compared to the wildfire group (M = 0.37, SD = 0.49 vs. M = 0.27, SD = 0.45; *t*_179_ = 1.69, *p* = 0.09), although, this difference was not significant. There was also no significant difference in suicidal ideation in the past month (M = 0.53 SD = 0.50 vs. M = 0.46, SD = 0.50; *t*_261_ = 0.97, *p* = 0.34).

In contrast to the findings for both depression and PTSD, there was no statistically significant between-group difference in terms of level of anxiety, although, the wildfire group exhibited slightly higher mean levels (M = 7.29, SD = 5.00 vs. M = 7.62, SD = 4.62; *t*_498_ = −0.97, *p* = 0.33). The three factors comprising the PTSD variable were not consistently affected, with the factors of re-experiencing (*t*_448_ = 3.48, *p* < 0.01) and avoidance (*t*_459_ = 3.53, *p* < 0.01) showing increased symptomatology in the prior trauma group, while no between-groups difference was observed for the hyperarousal factor (*t*_493_ = 1.00, *p* = 0.32).

Because a high number of individuals did not specify the nature of the trauma, the analysis was repeated excluding this group, to determine whether the pattern of statistical findings would be maintained. Using only specified trauma respondents, all of the significant effects from the initial analysis remained, but the hyperarousal factor (*t*_234_ = 3.00, *p* < 0.05) and lifetime suicide risk (*t*_99_ = 2.52, *p* < 0.05) were also significantly different.

### Examination of Potential Confounding Effects

To ensure these findings could not be better explained by confounding effects of age or sex, a one-way multivariate analysis of covariance (MANCOVA) was performed. The CPSS total score was removed from the analysis, due to its high correlations with its underlying factors, which were retained. Additionally, the suicide variables were dropped, as their inclusion resulted in undesirably low *n*'s in the overall model (*n*'s < 80).

The MANCOVA was performed with anxiety, depression, and the three PTSD variables (re-experiencing, avoidance, and hyperarousal) as the dependent variables, age, and sex as the covariates, weight, and two groups (prior trauma and wildfire only) of the independent variable. There was a statistically significant difference between the prior trauma and wildfire groups on these five dependent variables after controlling for age and sex, *F*_(5,951)_ = 9.394, *p* < 0.001, Pillai's Trace = 0.047, partial η^2^ = 0.047.

Univariate analyses revealed prior traumatization had a negative impact on depression *F*_(1,957)_ = 6.49, *p* = 0.011, partial η^2^ = 0.007, and two of the three PTSD variables: re-experiencing *F*_(1,957)_ = 18.94, *p* < 0.001, partial η^2^ = 0.019, and avoidance *F*_(1,957)_ = 13.85, *p* < 0.001, partial η^2^ = 0.014. Prior trauma was unrelated to either level of anxiety *F*_(1,957)_ = 0.24, *p* = 0.627, partial η^2^ = 0.000 or PTSD hyperarousal *F*_(1,957)_ = 1.68, *p* = 0.195, partial η^2^ = 0.002. Mean scores are displayed for the prior trauma and wildfire groups as a function of trauma type in Table 3.

**Table 3:** Means (SD) of mental health variables and MANCOVA results of differences between the prior trauma and wildfire groups, while controlling for age and sex.

**Mental health variable**	**Trauma Group**		***F*_**(1,957)**_**
	**Prior trauma group (*n =* 265)**	**Wildfire group (*n =* 694)**	
Anxiety	7.10 (4.84)	7.51 (4.64)	0.24
Depression	8.35 (6.85)	7.34 (5.92)	6.49^*^
PTSD—re-experiencing	2.94 (3.59)	2.16 (2.91)	18.94^**^
PTSD—avoidance	4.79 (5.23)	3.69 (4.48)	13.85^**^
PTSD—hyperarousal	4.61 (4.11)	4.44 (3.96)	1.68

* *p < 0.05*.

** *p < 0.01*.

### Subgroup Analysis

To more directly investigate the effect of retraumatization, subgroup analysis with MANCOVA, was also performed to examine effects of type of trauma on the mental health variables, while controlling again for age and sex. Analyses were conducted for anxiety, depression, and PTSD. Again, suicide was excluded from the analysis. As only four individuals reported physical abuse as their worst trauma, these individuals as well as those who did not specify the exact nature of the trauma were eliminated from further analysis.

There was a statistically significant difference in mental health scores as a function of the different trauma types, after controlling for both age and sex, *F*_(15,2499)_ = 6.752, *p* < 0.001, Pillai's Trace = 0.117, partial *n* = 0.039. Given the significance of the overall test, results of the dependent variables were considered separately. Univariate analyses revealed that trauma type had significant effects on all the mental health variables (all *F*'s > 7.00, *p*'s < 0.001).

*Post-hoc* tests using Bonferroni correction confirmed that the difference can be attributed to higher scores for survivors of sexual abuse, as compared to the other three categories of trauma (death, injury, and the wildfire itself). This was true for all mental health variables except PTSD re-experiencing, where there was also a significant difference between death of a loved one and the other two types (but sexual trauma was still highest). It appears that sexual abuse is associated with a statistically significant increase in mental health problems in retraumatized adolescents. Mean (SD) scores for the these mental health variables as a function of trauma type are displayed in Table 4.

**Table 4:** Means (SD) of mental health variables and MANCOVA results of differences between different types of trauma, while controlling for age and sex.

**Mental health variable**	**Trauma Type**				***F*_**(3,837)**_**
	**Wildfire (*n =* 694)**	**Death of someone close to you (*n =* 97)**	**Injury that you suffered (*n =* 22)**	**Sexual assault against you (*n =* 28)**	
Anxiety	7.51 (4.64)	7.30 (4.59)	5.86 (5.03)	11.61 (3.80)^*a*^	7.06^**^
Depression	7.34 (5.92)	8.28 (6.23)	7.86 (7.75)	15.18 (7.06)^*a*^	13.98^**^
PTSD—re-experiencing	2.16 (2.91)^*c*^	3.22 (3.51)^*b*^	2.23 (3.62)^*c*^	6.57 (4.09)^*a*^	24.27^**^
PTSD—avoidance	3.69 (4.48)	4.47 (4.49)	4.55 (4.82)	10.79 (5.64)^*a*^	22.61^**^
PTSD—hyperarousal	4.44 (3.96)	4.74 (3.78)	4.05 (4.53)	8.64 (3.65)^*a*^	10.72^**^

** *p < 0.01*.

*Italicized superscript letters indicate post-hoc analyses where one group differs statistically from the others*.

## Discussion

This study of retraumatization vs. newly acquired trauma suggests those who experienced prior trauma had higher rates of mental ill-health. In order to examine the effects of retraumatization, we examined the difference between the mental health of adolescents who had experienced a former traumatic event, compared to those whose first trauma was reported as the 2016 Fort McMurray wildfire. Individuals who experienced prior trauma had significantly higher rates of both depression and PTSD, but not in anxiety or risk of suicide. These results suggest a clear deleterious effect of previous trauma on current functioning, in the face of collective trauma.

With respect to PTSD, participants with previous trauma showed no difference in hypervigilance compared to the wildfire group; i.e., they were no more likely to confirm: *Having trouble concentrating* (1.06 [1.10] vs. 0.95 [1.02]); or *Having trouble falling or staying asleep* (1.13 [1.15] vs. 1.04 [1.13]). This is of interest because hypervigilance may be a sign of sympathetic activation (22). High rates of PTSD (37%) observed in adolescents even several years after the wildfire (23) remain both surprising and concerning as, for example, Bonanno's (24) review of chronic PTSD following experience of traumatic events suggests rates are typically much lower, between 6.6 and 17.8%. There maybe specific trajectories that put some individuals at increased risk for chronic distress (25). Persistent dysregulation has been described in cases of complex PTSD, in which trauma disrupts formative developmental periods (26). We suggest the high PTSD rates observed in this study could be partially mediated by undiagnosed retraumatization, with PTSD presenting differently between the groups.

There remains a need to explore a broader array of differential individual and contextual impacts (27) that may contribute to elevated rates of PTSD. While our rates were comparable to those observed in children and adolescents (28.6% of whom showed mild PTSD) following an Australian wildfire (28), that study looked at functioning 6 months post-event, in contrast to our data following 3.5 years. The influence of the ongoing, significant economic downturn Fort McMurray experienced following the wildfire should also be considered. In that period, a drop in the price of oil dramatically slowed economic recovery of the region, which had cumulative negative effects on a large proportion of the local population – many of whom worked directly in the oil and gas sector. This consequent widespread job loss accompanied a precipitous drop in housing prices. Those individuals willing to relocate to find employment elsewhere were paying for mortgages on homes they could no longer afford to sell, given low home valuations. This massive shift in circumstances meant that many adolescents in Fort McMurray endured the difficulties associated with evacuation and displacement due to the wildfire, only to return to face issues in terms of their parents' job loss and subsequent mental ill-health. The fact that nearly two-thirds (63.3%) of the current sample were excluded from analysis because they reported that their worst trauma had occurred *since* the wildfire supports this interpretation. It is worth noting that these kinds of contextual issues are particularly difficult for children, because most of these decisions are at the discretion of their caregivers, who ultimately make the decision as to whether or not to remain in the environment. This raises the question of whether young children may have a different profile compared to adolescents and young adults in this context, as young Canadian children typically have little or no sense of responsibility or control over their circumstances. One might speculate that, in order to return to pre-trauma levels of PTSD, adolescents require a return to a sense of normalcy in which they no longer feel a need to be “on their guard.” One possible interpretation is that, given dire economic and social circumstances that have been experienced since the wildfire, “across the board” normalcy has not yet returned to Fort McMurray. Ironically, wildfire remains a significant risk given increased temperatures and drier conditions due to climate change (29).

Retraumatization may look different in the context of a mass trauma event, such as the wildfire, compared to an individual-level stressor. First, some unknown proportion of individuals are at risk for retraumatization in these situations (in this study, nearly one-third), and it appears they may react differently and have poorer outcomes following a crisis. Based on our findings, we propose that retraumatization should be recognized as a risk factor in mass trauma situations. Programs and services must be truly trauma-informed to meet the needs of this vulnerable group. Second, mass traumatization can be influenced, in part, by processes of psychological contagion (30), with negative psychological impacts occurring through interactions with others who have experienced trauma. Stress contagion has been described as the presence of “behavioral (e.g., anxiety-like symptoms) and/or physiological (e.g., HPA-axis activation) sequelae of stress exposure… in those individuals who are not directly exposed to the stressor” (31). Our previous research on the impacts of the wildfire, which saw the evacuation of all 88,000 residents of Fort McMurray, demonstrated that a number of adolescents who were only “minimally impacted” (e.g., were not present in the town for the wildfire, and did not suffer impacts such as the loss of their home) still showed symptomatology consistent with PTSD (32). Stress contagion is comprised of the psychological (e.g., anxiety) and physiological changes that occur in response to exposure to stress, but it occurs in individuals not directly exposed to the stressor—only other affected individuals. A review of the contagion phenomenon (31) has reported instances between mother-infant dyads (33, 34) and in married couples (35, 36) but we believe our work represents the first reported instance of stress contagion occurring *en masse* as a result of collective trauma resulting form natural disaster. Research has attempted to identify potential neurochemical mechanisms that might act as proximal signals of distress (37), although, evidence of this kind of signaling taking place remotely [e.g., *via* videotape; (38)] potentially suggests a concomitant role for media exposure in mass trauma circumstances. Our results support the idea that spending time in that environment in the company of others who were significantly affected was, in itself, enough to increase trauma symptoms in some individuals.

Experience of mass trauma may also differ in terms of increased levels of social and family support (social buffering, rather than contagion). In situations of mass trauma, there is a collective experience and response, which often results in increased levels of empathy, and sharing of physical resources. This was witnessed during the evacuation in Fort McMurray, where individuals fleeing the town were met on the highway by others providing bottles of water and gasoline to those in need. In other words, being surrounded by other empathetic parties who have experienced the same trauma may actually carry some protective effect. These suppositions highlight the critical role of community support in building and strengthening resilience following mass crises such as natural disasters. If it is true that one of the main characteristics of trauma is that it is “fundamentally decontextualizing” with disconnection from others being a foregone result (39), then shared experience may be a key to improved coping skills amongst survivors. This is another way in which mass trauma differs from individual-level trauma, which is often characterized by feelings of isolation, loneliness, and exclusion. It is possible that the prior trauma group differed in terms of symptoms such as depression because they were not expecting those around them to understand or sympathize with their anxieties, as was likely their experience with their past individual-level trauma. At the same time, their prior trauma experience may have resulted in alterations in the likelihood of turning to others for support or assistance. In this context, it makes sense that the subgroup that suffered sexual abuse (as opposed to injury or death of a loved one) would experience the greatest coping difficulties, because sexual abuse consequences are often hidden and processed alone; it is possible these individuals had never learned to share their early feelings of grief and pain with others, and so did not see this as a potential coping response in the face of collective trauma. Interestingly, another paper examining retraumatization in veterans also found that sexual assault was the most robust predictor of increased risk for depression, PTSD, and suicidality (7). This is an issue worthy of continued investigation.

Finally, our results carry implications for educators and clinicians. This is particularly important in working with adolescents, as it has been suggested that the full effects of trauma may not be evident until adulthood (5). In the context of mass trauma, we should be sensitive to the fact that some individuals with a history of trauma may be more vulnerable to retraumatization. This reaffirms the need for services to be trauma-informed. However, in social situations where a mass trauma has occurred, it also suggests a need to treat the group as an ensemble, rather than reacting to specific individuals who are showing distress. Contagion studies suggest that those impacted react as a group and relationship should be central to therapeutic approaches (31, 40). In group settings, adolescents may work together to start finding ways to share the things they have found helpful, cognitively reframing the event, and incorporating what they have learned from the experience into a new worldview. This group dynamic therefore underscores the importance of leadership, with a role for teachers and therapists (who may themselves have also experienced the trauma) in helping adolescents develop the self-regulation skills promoting empathy among group members, as well as emotional maturity to recognize their own internal emotional distress signals.

## Limitations

There are several limitations of this study, which reduce its potential replicability and generalizability. One is that we were not able to collect information regarding the trajectory of the recovery post-wildfire. Data utilized for this analysis were collected 3.5 years after the 2016 wildfire; data gathered during or shortly after the wildfire might have looked quite different, as many factors likely changed in the interim. Being prepared for research in disaster situations of all types, in terms of readiness with a set of potential study tools to broadly measure physical and mental health, and a potential schedule of when this information might be gathered, would be useful so that deployment in these situations can be efficient and well-planned. Additionally, our decision not to track individual IDs (a tradeoff between reduced complexity/increased participation and ability to capture individual-level changes over time) was a definite limitation complicating our analysis.

Relatedly, while details of the difficult social and economic circumstances in Fort McMurray are well-documented (41)^1^, these variables were not measured directly. This represents another limitation in this study. It would have been useful to have an indication of how socioeconomic variables affected the overall recovery of the region post-wildfire, but at the time our focus was specifically on mental health, and there was little reason to believe at the time that the economic uncertainty was going to be long-term. The fact that nearly half of the adolescents surveyed reported that their greatest stressor had occurred in the years since the wildfire supports the supposition that this trauma was related to parental job loss, leading to financial instability, bankruptcy, mortgage foreclosure, etc., and other related issues, such as domestic conflict, including violence and marital breakdown, as reported anecdotally by local residents. Future studies should consider inclusion of direct measures of familial impacts, including economic well-being, and related mental health sequelae.

Another limitation of this study surrounds which adolescents were unavailable to take part in the study, and the fact that those taking part may not have been representative of the overall sample. Children who had left school (e.g., dropped out) or were simply not present that day would have missed it. This is noteworthy, as their absence could actually be indicative of poorer mental health. Children with a history of skipping school or leaving altogether may do so due to problems with handling the content, the social situation, or both. It is possible that these results represent an underestimate of difficulties. Additionally, because the survey was limited to students attending school within the Fort McMurray townsite, other adolescents living in nearby rural areas were excluded. Because this group of individuals includes Indigenous youth living on reserve, a significant segment of the local demographic, this dataset may not properly generalize to their experience.

There are other limits to the generalizability of this study, given the specific attributes of the townsite. Fort McMurray is a relatively small town (fewer than 100,000 residents) situated in a fairly remote, northern location. The nearest large city is over 4 h drive away, accessible by only one road—which held ramifications for the evacuation of the townsite. This means that residents are somewhat isolated and must be self-sufficient. It is also a very industrial, resource-dependent town. Prior to the fire and economic crisis, Fort McMurray was a net attractor for immigrants and internal migrants from other parts of Canada, due primarily to the potential for young people of making a very good income in the oil industry. For example, according to the 2016 Census^2^, the median total income for a family in Fort McMurray was $195,656 CAD; compare this to the median income in the neighboring province of Saskatchewan (which has a similar population density) at $75,412 CAD. This has resulted in a fairly young population (median age 33.1 years, compared to 37.8 years for Saskatchewan), with a bias toward males (54.0% compared to Saskatchewan's 49.7%). Finally, it is noteworthy, as discussed above, that the town went through a drastic reversal in fortunes in the years following the wildfire. All of these factors potentially limit the generalizability of these results to other populations.

There were also limitations in terms of the level of detail of the data gathered. For example, while we were able to ascertain which adolescents had experienced prior trauma, for the most part we grouped these types of trauma together due to sample size limitations. It should be recognized that different types of trauma (e.g., loss vs. abuse) are likely to have different impacts, as we note in our subanalysis. Similarly, it is also worth considering that chronicity of trauma (e.g., childhood abuse vs. a single-episode traumas) could similarly affect the degree or expression of retraumatization (5). To that end, it is worth considering that some of the prior trauma group may have had poorer mental health and been functioning at a lower overall level prior to the wildfire. Again, due to the design of this study, such data could not be collected. Ideally, a prospective study having baseline measures of functioning for individuals with these types of underlying trauma would be useful in the future.

Finally, because social effects appear central to coping with a mass trauma (for both better and worse), future investigations should try to capture not only the perceptions of the affected individual, but his or her impressions of how those around them are coping. Our study did not attempt to capture this type of information. In mass trauma events, where the common understanding is that “everyone is going through the same thing,” discrepancies in terms of resources and attitudes may in fact cause some individuals to feel more isolated, which could have broader mental health repercussions.

## Conclusion

Retraumatization needs to be identified as an underlying, often unrecognized vulnerability that may worsen health outcomes for some individuals in the event of a mass trauma. In this study, nearly one-third of the population fell into this group. For this reason, it is important that those offering healthcare and social services in mass crisis situations ensure that the services they deliver are truly trauma-informed, to provide the best possible outcomes for those most vulnerable to retraumatization. Based on the limitations of this study, we would suggest that, in the event of a mass trauma, it is important for research teams to be prepared and proactive to evaluate these needs. Although, early measurements can be challenging as individuals actively deal with a crisis, these data may be invaluable in understanding the trajectory of change in recovery. Measurements should include basic indicators of physical and mental health, but also track socioeconomic factors, which can themselves affect group functioning. Finally, this study underscores the value of assessing individuals for prior trauma, to help identify which individuals may be in need of additional assistance in disaster situations. That said, mass trauma events also offer an opportunity for community members to support one another in ways that individual-level traumas may not, because there is a sense that everyone is in it together, and individuals may be able to lean on one another for support. Until we better understand the scope and overall effects of undiagnosed trauma in the population, we will fail to meet the needs of this vulnerable group.

## Data Availability Statement

The data analyzed in this study is subject to the following licenses/restrictions: data for this study is the property of the Fort McMurray Public and Catholic School Districts. Requests to access these datasets should be directed to Matthew R. G. Brown, mrbrown23@gmail.com.

## Ethics Statement

The studies involving human participants were reviewed and approved by Health Research Ethics Board, University of Alberta. Written informed consent to participate in this study was provided by the participants' legal guardian/next of kin.

## Author Contributions

HP, MB, and PS: study design and analysis. HP, MB, SN, MM, and DK: data collection. HP, MB, CM-H, AG, VA, BL, JD, JO, PB-M, and PS: manuscript preparation. All authors contributed to the article and approved the submitted version.

## Conflict of Interest

The authors declare that the research was conducted in the absence of any commercial or financial relationships that could be construed as a potential conflict of interest.

## Footnotes

^1^https://financialpost.com/commodities/energy/from-binge-to-bust-a-canadian-oil-town-lines-up-at-the-food-bank

^2^https://www12.statcan.gc.ca/census-recensement/2016/dp-pd/prof/details/page.cfm?Lang=E&Geo1=POPC&Code1=0292&Geo2=PR&Code2=47&Data=Count&SearchText=FortMcMurray&SearchType=Begins&SearchPR=01&B1=All&wbdisable=true
